# Role of Endothelial Progenitor Cells and Inflammatory Cytokines in Healing of Diabetic Foot Ulcers

**DOI:** 10.1371/journal.pone.0083314

**Published:** 2013-12-16

**Authors:** Francesco Tecilazich, Thanh Dinh, Leena Pradhan-Nabzdyk, Ermelindo Leal, Ana Tellechea, Antonios Kafanas, Charalambos Gnardellis, Mary L. Magargee, Andre Dejam, Vasilis Toxavidis, John C. Tigges, Eugenia Carvalho, Thomas E. Lyons, Aristidis Veves

**Affiliations:** 1 Microcirculation Laboratory and Joslin-Beth Israel Deaconess Foot Center, Beth Israel Deaconess Medical Center, Harvard Medical School, Boston, Massachusetts, United States of America; 2 Center for Neurosciences and Cell Biology, University of Coimbra, Coimbra, Portugal; 3 Technological Educational Institute of Messolonghi, Messolonghi, Greece; 4 Division of Cardiology, Beth Israel Deaconess Medical Center, Harvard Medical School, Boston, Massachusetts, United States of America; 5 Flow Cytometry Core Facility, Beth Israel Deaconess Medical Center and Harvard Stem Cell Institute, Boston, Massachusetts, United States of America; University Hospital Hamburg-Eppendorf, Germany

## Abstract

**Background:**

To evaluate changes in endothelial progenitor cells (EPCs) and cytokines in patients with diabetic foot ulceration (DFU) in association with wound healing.

**Methods:**

We studied healthy subjects, diabetic patients not at risk of DFU, at risk of DFU and with active DFU. We prospectively followed the DFU patients over a 12-week period. We also investigated similar changes in diabetic rabbit and mouse models of wound healing.

**Results:**

All EPC phenotypes except the kinase insert domain receptor (KDR)^+^CD133^+^ were reduced in the at risk and the DFU groups compared to the controls. There were no major EPC differences between the control and not at risk group, and between the at risk and DFU groups. Serum stromal-cell derived factor-1 (SDF-1) and stem cell factor (SCF) were increased in DFU patients. DFU patients who healed their ulcers had lower CD34^+^KDR^+^ count at visits 3 and 4, serum c-reactive protein (CRP) and granulocyte-macrophage colony-stimulating factor (GM-CSF) at visit 1, interleukin-1 (IL-1) at visits 1 and 4. EPCs tended to be higher in both diabetic animal models when compared to their non-diabetic counterparts both before and ten days after wounding.

**Conclusions:**

Uncomplicated diabetes does not affect EPCs. EPCs are reduced in patients at risk or with DFU while complete wound healing is associated with CD34^+^KDR^+^ reduction, suggesting possible increased homing. Low baseline CRP, IL-1α and GM-CSF serum levels were associated with complete wound healing and may potentially serve as prognostic markers of DFU healing. No animal model alone is representative of the human condition, indicating the need for multiple experimental models.

## Introduction

Diabetic foot ulceration (DFU) is a major health problem with considerable morbidity and mortality along with heavy financial burden for health care services [1]. Although neuropathy, vascular disease and trauma are the main factors that lead to the development of DFU, impaired wound healing is the main factor that leads to development of chronic wounds and lower extremity amputations [2]. Previous studies in our unit have shown that pre-existing inflammation and aberrant growth factor levels are directly associated with failure to heal an ulcer [3]. 

Bone marrow-derived endothelial progenitor cells (EPCs) play an important role in angiogenesis and in maintaining the integrity and function of vascular endothelium, as they are mobilized either in response to tissue ischemia or by various cytokines to integrate into new and existing blood vessels [4,5]. Initial studies showed severely reduced EPC levels in diabetic patients with peripheral arterial disease (PAD) and patients with diabetic vascular complication especially in patients with foot lesions [6,7]. Recent studies reported reduced EPC levels in diabetes irrespective of the presence or absence of macrovascular disease while the presence of macrovascular disease in non-diabetic subjects did not affect EPC levels [8]. Although the role of EPCs in DFU healing in human subjects has not been explored, preliminary studies in diabetic patients with critical limb ischemia (CLI) indicated that autologous transplantation of granulocyte colony-stimulating factor (G-CSF)-mobilized peripheral blood mononuclear cells (PBMNCs) improved blood flow and led to complete wound healing [9]. 

The main aim of the present study was to evaluate changes that occur in EPCs, growth factors and cytokines in patients with active DFU and their association with the progression of wound healing. In order to achieve this, we first conducted a cross-sectional study that compared differences in four groups: 1) healthy control subjects, 2) diabetic patients without serious complications, 3) diabetic patients at risk of DFU in the absence of peripheral arterial disease, and 4) diabetic patients with DFU. We also conducted a prospective study that followed the DFU patients over a 12-week period and examined the progress in wound healing in relationship to changes in EPC numbers and cytokine expression. We finally extended the investigation into rabbit and mouse models of diabetic wound healing.

## Research Design and Methods

### Subjects

All research subjects were recruited from patients who attended the Joslin-Beth Israel Deaconess Foot Center. We studied four groups: healthy control subjects; diabetic patients not at risk of DFU; patients with peripheral neuropathy severe enough to put them at risk of DFU according to previously published criteria [10]; and diabetic patients with chronic DFU at the forefoot (defined as a clinically non-infected ulcer that was present for at least four weeks and extended through the dermis and into subcutaneous tissue but without exposure bone or joint capsule). Exclusion criteria were: Clinically present PAD, end stage renal disease, active Charcot neuroarthropathy, severe heart failure and/or any other serious illnesses. 

We also examined discarded skin specimens of age and sex matched healthy, non-diabetic subjects and diabetic patients with peripheral neuropathy who underwent foot surgery. Seven healthy, non-diabetic subjects [age 55 ±18 years (mean±sd), 3 males] and 10 diabetic patients (61±10 years, 6 males) were included. The study was approved by the IRB of the Beth Israel Deaconess Medical Center (2006-P-000335) and all participants signed informed consent.

### Methods

#### Baseline visit

Subjects in the first three groups were seen once while DFU patients were followed for twelve weeks and were seen for a total of 3 additional visits every four weeks. DFU treatment was provided according to standard guidelines [11]. 

#### Clinical and laboratory assessments

The measurements of inflammatory cytokines and growth factors were performed with a Luminex 200 apparatus (Luminex Corporation Austin, TX) and Millipore multiplex immunoassay panels (Millipore Corporation, Chicago, IL). 

#### Evaluation of Diabetic Neuropathy and Peripheral Arterial Disease

Neuropathy symptoms were evaluated by using a Neuropathy Symptom Score (NSS), clinical signs by using a Neuropathy Disability Score (NDS), assessment of Vibration Perception Threshold and Cutaneous Perception Threshold using a set of twelve Semmes-Weinstein monofilaments ranging from 2.83 to 10 g. Patients who were defined at high risk of foot ulceration had NDS≥5 and were unable to feel a 5.07 Semmes-Weinstein monofilament [10]. Peripheral Arterial Disease was assessed clinically; patients with Ankle Brachial Index (ABI) ≤0.7, absence of foot pulses and/or claudication were excluded from the study.

#### Vascular Reactivity Tests

The vascular reactivity of the forearm skin microcirculation was evaluated by Laser Doppler perfusion imaging measurements before and after the iontophoresis with acetylcholine chloride (Ach, endothelium-dependent vasodilation) and sodium nitroprusside (SNP, endothelium-independent vasodilation) as previously described [12]. The Nerve Axon Reflex-Related Vasodilation was performed by using a single point Laser Probe and the Moor DRT4 System [12]. The flow mediated brachial artery dilation (FMD, endothelium dependent) and nitroglycerine-induced dilation (NID, endothelium independent) were measured in accordance with published guidelines [13]. 

#### Evaluation of Oxy- and Deoxy-Hemoglobin

Data were collected with a HyperMed System (HyperMed, Inc., Burlington, MA) as previously described [14]. 

#### Flow cytometry measurements of EPCs

**Figure 1:**
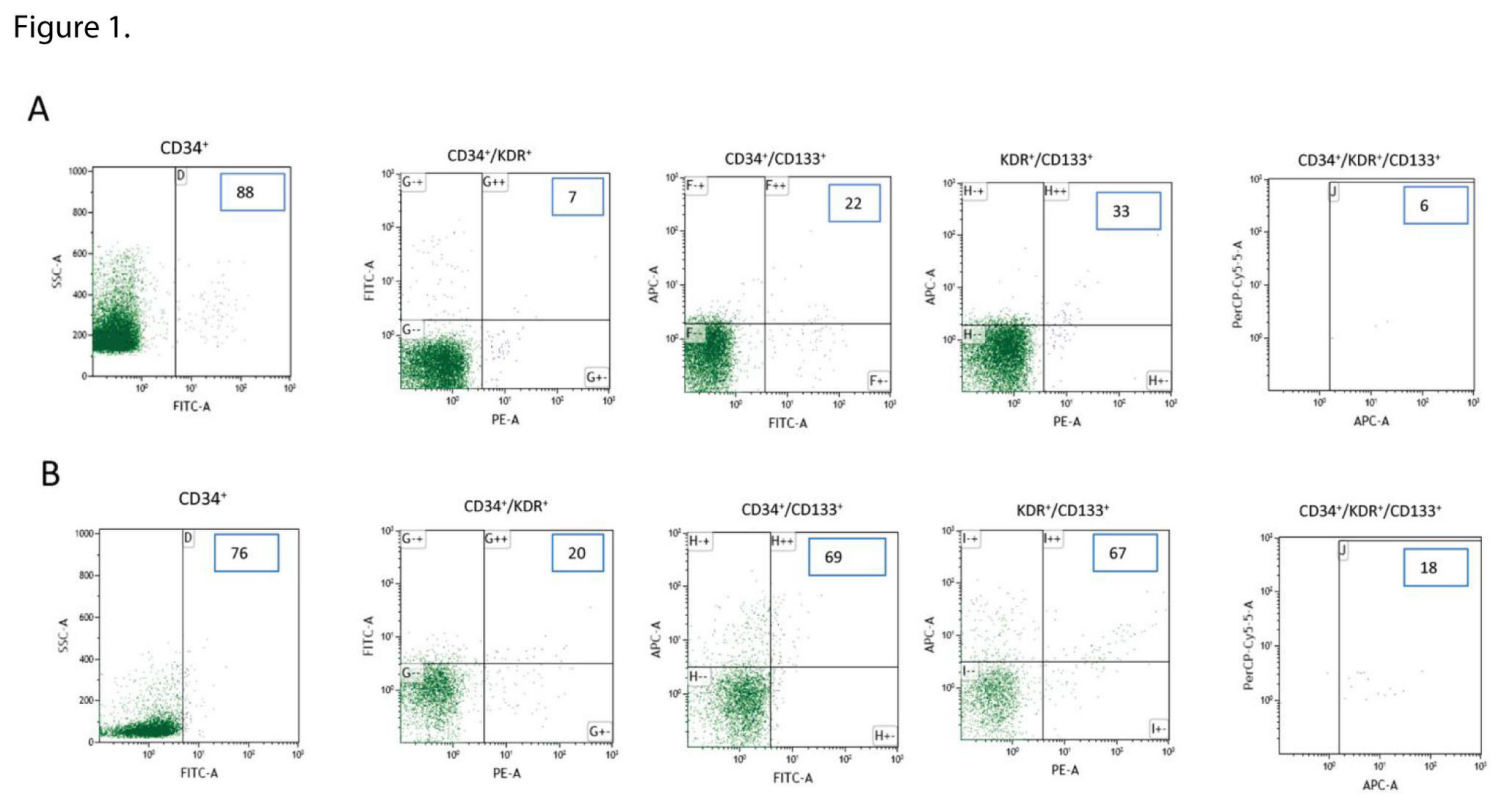
An example of flow cytometric analysis of human peripheral blood sorted on CD45^dim^ cells in a patient with DFU (A) and a healthy control subject (B). The triple positive phenotype (CD34^+^/KDR^+^/CD133^+^) was determined by gating the CD133^+^ cells on the CD34^+^/KDR^+^. 1.000.000 events per sample were acquired and the counts for each phenotype are shown in the picture. Smaller counts in all phenotypew were observed in the diabetic patient (A) when compared to the healthy subject (B) in the double and triple measurements.

The measurements of the various EPC phenotypes were performed at the Beth Israel Deaconess Flow Cytometry Core Facility. Immunofluorescent cell staining was performed on peripheral blood with the use of the fluorescent conjugated antibody CD34–fluorescein isothiocyanate (FITC, Becton Dickinson), type 2 vascular endothelial growth factor receptor 2 (VEGF-R2)/KDR (kinase insert domain receptor)-phycoerythrin (PE, Miltenyi Biotec), CD133-allophycocyanin (APC, Miltenyi Biotec) and CD45-Peridinin Chlorophyll Protein (PerCP, Miltenyi Biotec) according to standard techniques. We employed a hierarchical gating strategy to count negative or low expressing CD45 cells (CD45^dim^). 1.000.000 events per sample were acquired using a FACS LSR II analyzer (Becton Dickinson, Franklin Lakes, NJ, USA) and the results were analyzed using the Beckman Coulter Kaluza analysis software (Beckman Coulter Inc., Brea, CA, USA). An example of measurements in a patient with DFU and a healthy control subject are presented in Figure 1. The same methods were employed for the animal models.

#### Forearm Skin Biopsies

One two-millimeter forearm skin punch biopsy was taken from a different cohort of control and diabetic subjects as previously described [3]. 5μm frozen sections were immunostained with anti-human stromal cell derived factor-1 (SDF-1, 1:50; Abcam, Cambridge, MA, USA) and anti-human CXCR4 (1:100; Chemicon International, Temecula, CA). A semi-quantitative analysis was performed for intensity (grade: 1=faint ; 2=low; 3=medium; 4=strong). The presence of staining in stromal cells (grade: 1 for 5-30% of cells expressing the antibody; 2 for 30-60%; 3 for more than 60%) and in endothelial cells (grade: 1 for no stain; 2 for positive endothelial cells staining) was also evaluated.

#### Animal Models

***Mice***: Diabetes was induced in 12-week old C57BL6/J mice with 50 mg/kg STZ intraperitoneally for 5 consecutive days) in citrate buffer (0.1M), while non-diabetic controls received vehicle alone. Fasting blood glucose was monitored one week post treatment, and mice with blood glucose over 250 mg/dL were considered diabetic. After 8 weeks, two 6 mm excisional wounds were createdn the shaved skin dorsum of the animals. Wound healing progress was measured daily by acetate tracing during 10 days. 

***Rabbits:*** Rabbits were made diabetic by alloxan (75mg/kg) to achieve fasting blood glucose over 250 mg/dL. Ten days after induction of diabetes, full thickness 6mm punch biopsy circular wounds were created in rabbit ears as previously described [15]. Wound healing was monitored over a ten day period. All animal studies were approved by the Beth Israel Deaconess Medical Center Institutional Animal Care and Use Committee (IACUC).

#### Data analysis

The Minitab statistical package (State College, PA) was used. For parametrically distributed data, the analysis of variance test was used, followed by the Fisher test to identify differences among the various groups. For nonparametrically distributed data, the Kruskal - Wallis test was used. For the comparison in changes during the four visits of the prospective study, the Friedman test was employed. The Spearman correlation coefficient *r* was used for the correlation of different parameters.

## Results

### Comparisons among the four tested groups

We recruited 131 subjects: 29 healthy control subjects, 39 diabetic (DM) patients not at risk of DFU, 25 DM patients at risk of DFU and 29 patients with an active DFU ( Table 1). The foot nerve axon reflex-related vasodilation, a measurement of the c-nociceptive fibers function, was reduced in all diabetic subjects when compared to the controls and was further reduced in the DFU group when compared to the other two diabetic groups (p<0.0001). The vasodilatory response to acetylcholine, a measurement of the foot skin endothelium function, was reduced in the diabetic patients at risk of DFU (p<0.05). There were no differences in the dorsum foot skin oxy- and deoxyhemoglobin and the saturated oxyhemoglobin among the four groups. FMD was reduced in the DM patients at risk of DFU and the DFU patients when compared to the other two groups (p<0.0001). The differences in serum growth factors and inflammatory cytokines are shown in Table 2. DFU patients had higher levels of growth related oncogene (GRO, p<0.02), interleukin-8 (IL-8, p<0.01), macrophage-derived chemokine (MDC, p<0.02), tumor necrosis factor alpha (TNFα, p<0.0001), c-reactive protein (CRP, p<0.01), SDF-1 (p<0.0001) and stem cell factor (SCF, p<0.0001). 

**Table 1:** Comparisons in subject demographics among the four tested groups using the Analysis of Variance (ANOVA) test for parametrically distribuited data and the Kruskal - Wallis test for nonparametrically distributed data.

	**Controls(C)**	**DM-not at risk of DFU (NR)**	**DM- at risk of DFU (R)**	**DM-DFU(DFU)**
No	38	39	25	29
Age (years)	58 ± 10	62 ± 11	62 ± 9	56 ± 9
Males (%)	24 (63)	22 (56)	16 (64)	23 (79)
BMI ^a^	26.9 ± 7.1	31.5 ± 5.2	31.1 ± 6.4	33.5 ± 5.7
DM Type (1/2)	-	8/31	7/18	8/21
DM Duration (years) ^b^	-	15 ± 14	26 ± 16	15 ± 9
HbA1c c (%) ^c^SI, IFCC-recommended units (mmol/mol)	5.7 ± 0.439 ± 4.4	7.0 ± 0.753 ± 7.7	7.5 ± 1.258 ± 13.1	8.6 ± 2.270 ± 24.0
Fasting Blood Glucose (mg/dl) ^c^	88 ± 10	116 ± 36	124 ± 52	168 ± 95
Systolic Blood Pressure (mmHg) ^d^	121 ± 15	120 ± 12	129 ± 17	127 ± 14
Diastolic Blood Pressure (mmHg)	71 ± 9	72 ± 9	72 ± 10	71 ± 11
Creatinine (mg/dl) ^e^	0.9 ± 0.2	1.0 ± 0.3	1.3 ± 0.4	1.3 ± 0.5
Total Cholesterol ^f^	200 ± 37	166 ± 29	153 ± 44	174 ± 41
HDL Cholesterol ^g^	62 ± 20	57 ± 18	45 ± 15	50 ± 10
LDL Cholesterol ^a^	112 ± 31	84 ± 21	79 ± 31	92 ± 34
Triglycerides	143 ± 88	135 ± 75	153 ± 97	161 ± 87
Neuropathy Symptom Score (NSS) ^h^	0 ± 1	2 ± 3	5 ± 4	5 ± 4
Neuropathy Disability Score (NDS) ^i^	0 ± 0	2 ± 2	13 ± 6	15 ± 6
Vibration Perception Threshold (volts) ^i^	8 ± 4	16 ± 10	39 ± 15	44 ± 12
Semmes-Weinstein Monofilaments ^h^	3.93 ± 0.28	4.24 ± 0.61	5.95 ± 1.26	6.46 ± 0.76
Foot Nerve Axon Reflex-Related Vasodilation (NARV) (%) ^j^	40 (20 : 81)	16 (1 : 49)	17 (3 : 41)	5 (-8 : 12)
Foot Endothelium Dependent Vasodilation (ACh response) (%) ^k^	28 (12 : 43)	24 (8 : 47)	11 (5 : 22)	20 (5 : 35)
Foot Endothelium Independent Vasodilation (SNP response) (%)	25 (16 : 53)	33 (19 : 62)	21 (8 : 34)	20 (6 : 51)
Dorsum Foot Oxy-Hb (AU)	32 (22 : 45)	41 (25 : 53)	29 (23 : 42)	36 (25 : 55)
Dorsum Foot Deoxy-Hb (AU)	48 (32 : 65)	53 (43 : 62)	46 (34 : 57)	47 (37 : 60)
Dorsum Foot O_2_ Hb Saturation (%)	42 (33 : 48)	39 (31 : 58)	42 (31 : 51)	45 (38 : 53)
Flow Mediated Vasodilation (FMD, %) ^e^	7.5 ± 2.5	6.7 ± 2.4	4.2 ± 2.1	4.9 ± 2.2
Nitroglycerin Induced Vasodilation (NID, %) ^l^	15.3 ± 5.7	14.9 ± 4.0	11.2 ± 5.5	13.9 ± 4.0
Ankle Brachial Index	%1.1 ± 0.1	1.0 ± 0.1	1.1 ± 0.2	1.1 ± 0.2
Statin [n, (%)] ^a^	7 (18%)	26 (67%)	18 (72%)	16 (55%)
Angiotensin Converting Enzyme inhibitors / Angiotensin II Receptor Blocker [n, (%)] ^m^	3 (8%)	17 (44%)	13 (52%)	20 (69%)
Insulin [n, (%)] ^n^	--	15 (38%)	16 (64%)	24 (83%)
Oral hypoglycemic [n, (%)]	--	23 (59%)	9 (36)	10 (35)

Mean ± sd or median (25-75 interquartiles)

a: C vs. NR, R, U: p<0.0001; b: R vs. NR, U: p<0.01; c: C vs. NR, R, U; U vs. NR, R: p<0.0001; d: C vs R; NR vs R, U: p<0.05; e: C, NR vs. R, U: p<0.0001; f: C vs. NR, R, U; R vs. U: p<0.0001; g: C vs. R, U; NR vs. R: p=0.001; h: C vs. NR, R, U; NR vs. R, U: p<0.0001; i: C, NR vs. R, U; R vs. U: p< 0.0001; j: C vs. NR, R, U; U vs. NR, R: p< 0.0001; k: C vs. R: p<0.05; l: C vs. R, U; NR vs. R: p<0.02; m: C vs. NR, R, U; NR vs. U: p<0.0001; n: NR vs. R, U: p=0.001

**Table 2:** Comparisons in flow cytometry, growth factor and cytokine results among the four tested groups.

	**Controls(C)**	**DM-not at risk of DFU (NR)**	**DM- at risk of DFU(R)**	**DM-DFU(DFU)**
CD34^+ a^	904 (240 : 3008)	825 (289 : 2128)	160 (75 : 345)	172 (59 : 740)
CD34^+^KDR^+ b^	129 (83 : 479)	91 (39 : 225)	27 (19 : 38)	42 (20 : 175)
CD34^+^CD133^+ c^	117 (48 : 372)	104 (16 : 163)	30 (18 : 43)	61 (15 : 133)
KDR^+^CD133^+^	52 (13 : 175)	45 (7: 158)	25 (12 : 35)	36 (10 : 93)
CD34^+^KDR^+^CD133^+ d^	18 (6 : 55)	11 (4 : 24)	6 (3 : 11)	3 (2 : 8)
Platelet-Derived Growth Factor-AA (ng/ml) ^e^	5.9 (3.7 : 7.4)	6.1 (4.0 : 8.8)	4.3 (2.8 : 6.5)	7.7 (4.5 : 13.7)
Platelet-Derived Growth Factor- AB-BB (ng/ml)	36.7 (22.8 : 67.3)	43.6 (22.7 : 75.9)	19.8 (10.3 : 60.0)	50.9 (25.8 : 83.5)
EGF (pg/ml)	37.0 (16.0 : 55.5)	41.0 (24.5 : 118.0)	28.0 (12.0 : 47.0)	36.5 (14.0 : 99.3)
Fibroblast Growth Factor-2 (pg/ml)	17.1 (7.6 : 37.7)	17.2 (8.2 : 35.0)	16.6 (9.7 : 48.1)	20.0 (18.9 : 38.2)
VEGF (pg/ml)	173 (92 : 268)	150 (117 : 211)	161 (47 : 266)	185 (103 : 253)
Insulin (pg/ml) ^f^	415 (228 : 698)	576 (284 : 1016)	1190 (646 : 1948)	468 (256 : 957)
Leptin (ng/ml)^g^	7.7 (2.9 : 17.1)	9.0 (4.3 : 25.9)	18.0 (8.5 : 41.8)	13.1 (7.3 : 30.5)
G-CSF (pg/ml)	30.7 (15.8 : 42.0)	32.1 (22.5 : 49.7)	36.1 (23.2 : 45.4)	32.7 (25.2 : 43.5)
GM-CSF (pg/ml)	1.7 (1.2 : 4.5)	1.6 (1.2 : 4.5)	3.81 (0.85 : 17.3)	2.3 (1.5 : 3.5)
Eotaxin (pg/ml)	139 (90 : 199)	177 (127 : 216)	149 (91 : 206)	167 (107 : 202)
GRO (pg/ml)^h^	565 (377 : 967)	665 (499 : 928)	531 (335 : 669)	840 (618 : 1261)
IFN γ (pg/ml)	1.72 (1.28 : 5.97)	3.53 (1.69 : 7.99)	2.20 (0.40 : 10.40)	2.45 (1.72 : 5.49)
Interleukin-6 (pg/ml)	4.38 (0.83 : 16.02)	2.56 (1.53 : 10.78)	5.94 (1.48 : 10.02)	7.15 (3.61 : 13.90)
IL-8 (pg/ml)^i^	13.6 (6.6 : 23.7)	15.7 (7.0 : 33.0)	11.0 (6.3 : 21.3)	29.5 (17.1 : 57.6)
Monocyte Chemoattractant Protein-1(pg/ml) ^k^	464 (336 : 612)	535 (414 : 694)	412 (298 : 602)	605 (444 : 784)
MDC (pg/ml)^l^	1326 (1047 : 1630)	1402 (1027 : 1683)	1320 (966 : 1720)	1667 (1370 : 1962)
TNFα (pg/ml)^m^	9.2 (4.5 : 11.3)	9.3 (6.0 : 14.2)	9.9 (7.5 : 18.3)	14.6 (10.8: 22.0)
MMP-9 (ng/ml)^n^	291 (182 : 402)	331 (212 : 471)	249 (190 : 302)	377 (272 : 525)
E-Selectin (ng/ml)	45.0 (32.8 : 58.1)	48.5 (36.5 : 64.3)	51.0 (37.0 : 63.8)	38.9 (26.2 : 55.1)
ICAM (ng/ml)	120 (96 : 159)	140 (118 : 165)	130 (114 : 209)	141 (109 : 170)
VCAM (ng/ml)	1039 (943 : 1318)	1150 (922 : 1291)	1151 (1054 : 1363)	1202 (1016 : 1636)
CRP (μg/ml)^o^	5.6 (1.7 : 14.7)	2.1 (1.2 : 18.8)	7.1 (1.8 : 22.8)	13.0 (5.0 : 53.7)
SDF-1 (pg/ml)^m^	4.73 (3.0 : 6.04)	4.39 (3.21 : 5.86)	4.12 (3.03 : 5.47)	7.60 (5.20 : 10.92)
SCF (pg/ml)^p^	11.1 (5.5 : 21.1)	4.4 (0.9 : 11.5)	15.8 (7.3 : 37.6)	37.4 (12.5 : 63.0)

As the data was nonparametrically distributed the Kruskal - Wallis test was employed.

Median (25-75 interquartiles), flow cytometry results are presented as events/10^6^

a: C, NR vs. R,U, p<0.0001; b: C vs. R, U; R vs. NR, U: p<0.0001; c: C vs. R, U; NR vs. R: p<0.005; d: C vs. R, U; NR vs. U: p=0.001; e: U vs. C, R; R vs. NR: p< 0.02; f: R vs. C, NR, U: p<0.01; g: C vs. R, U; R vs. NR: p<0.02; h: U vs. C, R: p<0.02; i: U vs. C, NR, R: p<0.01; k: R vs. U, NR: p<0.05; l: U vs. C, NR, R: p=0.02; m: U vs. C, NR, R: p<0.0001; n: R vs. U, NR: p=0.02; o: U vs. C, NR, R: p<0.01; p: C vs. U; NR vs. C, R, U: p<0.0001

For technical reasons, reliable flow cytometry results were available in 25 controls, 28 DM patients not at risk of DFU, 20 DM patients at risk of DFU and 25 DM patients with DFU (Figure 1). There were no major differences between the control subjects and the not at risk of DFU group (Table 2). In addition, there were no major differences between the at risk of DFU and the DFU groups except the CD34^+^KDR^+^ phenotype was reduced in the at risk of DFU group. Compared to the controls, all tested EPC phenotypes except for the KDR^+^CD133^+^ were reduced in the at risk of DFU and the DFU groups. Patients not at risk of DFU also had higher counts of most EPC phenotypes when compared to the other two diabetic groups even though the differences were not as prominent as the ones observed with the control group. 

The relationship between EPC measurements, FMD and biochemical measurements of inflammation and endothelial dysfunction was evaluated by performing correlation and evaluating the correllation coefficient r. For this, all subjects in all four groups were considered as one group. Positive correlations were observed between FMD and all flow cytometry measurements (CD34^+^: r=0.32, p<0.01, CD34^+^KDR^+^: r=0.31, p<0.01, CD34^+^CD133^+^: r=0.26, p<0.05, KDR^+^CD133^+^: r=0.28, p<0.01, CD34^+^KDR^+^CD133^+^: r=0.31, p<0.05). Positive correlations were also observed between HDL and KDR^+^CD133^+^ (r=0.48, p<0.0001) and CD34^+^KDR^+^CD133^+^ (r=0.22, p<0.05). A negative correlation existed between CD34^+^ and TNFα (r= -0.22, p<0.05), while strong positive correlations were observed between KDR^+^CD133^+^ and serum epidermal growth factor (EGF, r=0.33, p<0.05), E-Selectin (r=0.67, p<0.0001) and intercellular adhesion molecule-1 (ICAM, r=0.42, p<0.0001). No correlations were observed between HbA1c levels and EPC measurements.

### Follow up results in the patients with DFU

**Figure 2:**
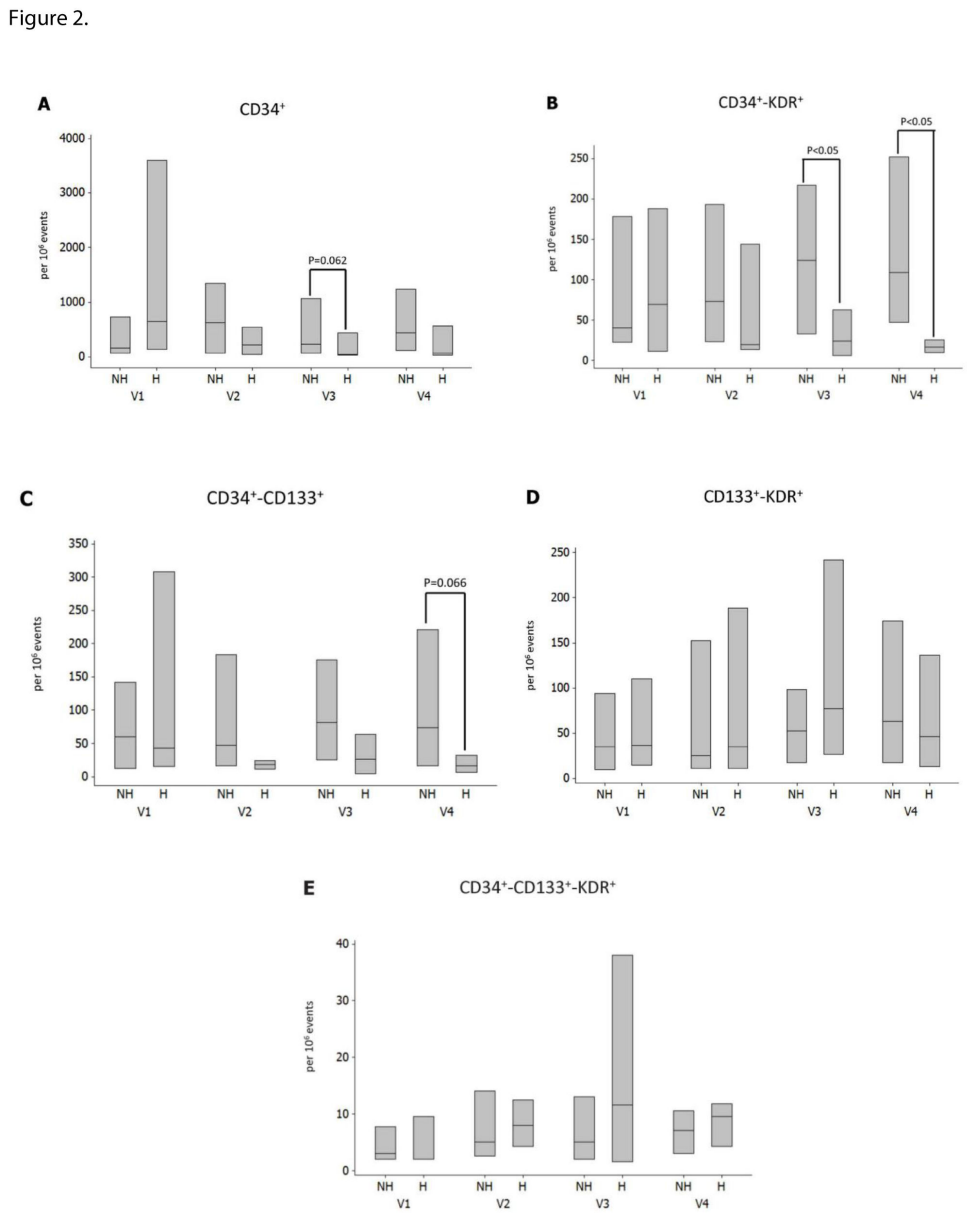
Changes in EPCs measurements during the four study visits between the patients who did not heal their ulcers (NH) and those who did (H). There were no differences in all EPC measurements at baseline but patients who healed their ulcers had lower CD34^+^KDR^+^ counts at visits 3 and 4 and CD34^+^CD133^+^ at visit 4. Data are presented as the median and interquartile range box.

**Figure 3:**
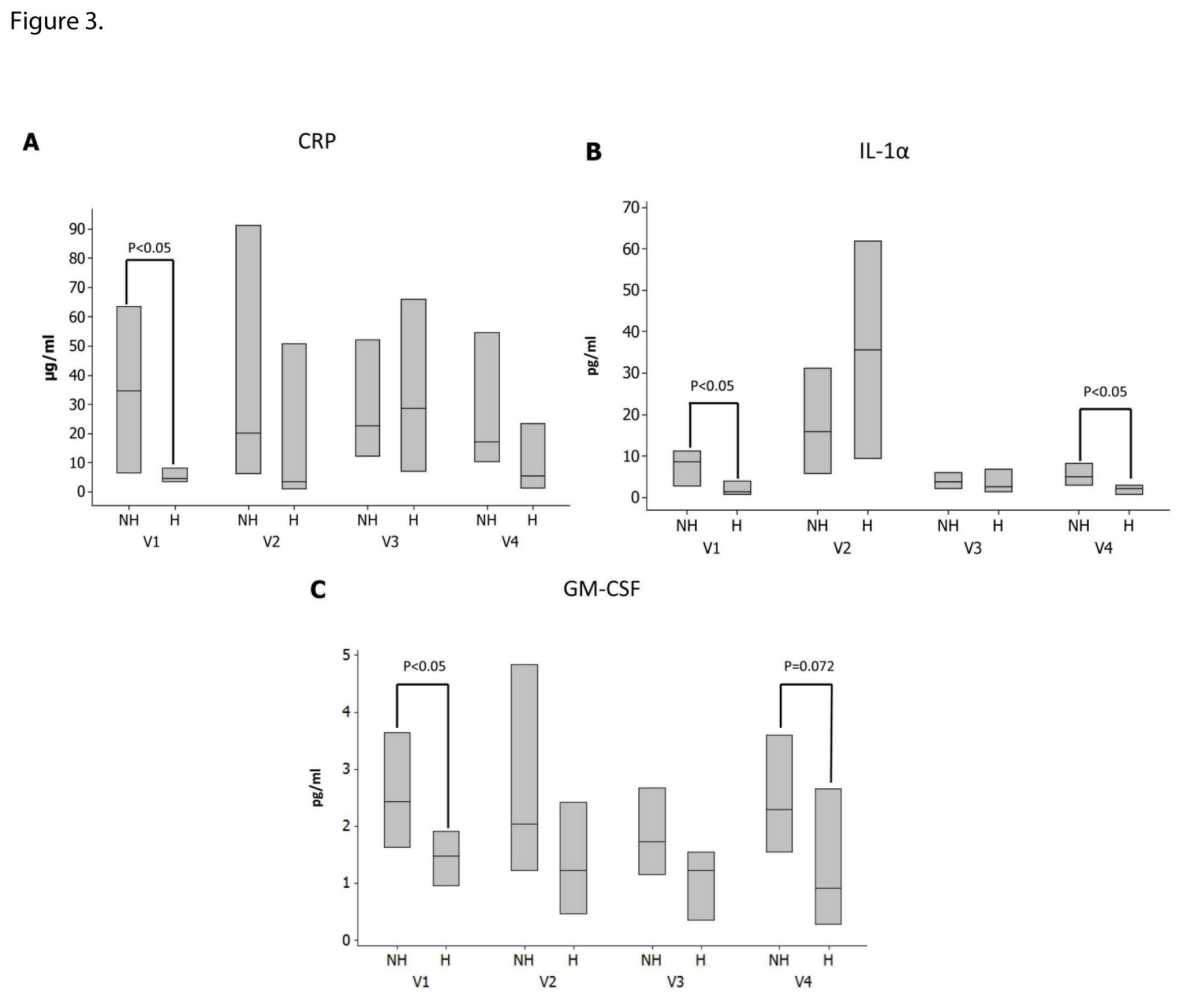
Changes in CRP (3a), IL-1a (3B) and GM-CSF (3C) during the four study visits between the patients who did not heal their ulcers (NH) and those who did (H). Data are presented as the median and interquartile range box. Patients who healed their ulcers had lower serum CRP and GM-CSF at visit 1 and lower IL-1α at visits 1 and 4.

The patients with DFU were seen four times every four weeks for a total period of 12 weeks. Complete wound healing was achieved in four patients during this period while two more patients completely healed in the three months after the exit visit. The most important differences between the patients who completely healed their ulcers during the study period and those who did not, are shown in Figures 2 and 3. Thus, although there were no differences in all EPC measurements at baseline, patients who healed their ulcers had lower CD34^+^KDR^+^ counts at visits 3 and 4 (Figure 2B, p<0.05), marginally lower CD34^+^ at visit 3 (Figure 1A, p=0.062) and CD34^+^CD133^+^ at visit 4 (Fig, 2C, p=0.066). Patients who healed their ulcers also had lower serum CRP at visit 1 (Figure 3A, p<0.05), lower interleukin-1 alpha (IL-1α) at visit 1 (Figure 3B, p<0.05) and visit 4 (p<0.05), lower Granulocyte-Macrophage Colony-Stimulating Factor (GM-CSF) at visit 1 (Figure 3C, p<0.05) and marginally lower GM-CSF at visit 4 (p=0.072).

The flow cytometry, serum growth factors and cytokine measurements in all DFU patients, irrespective of the healing outcome, at all four visits during the 12-week study period are summarized in Table 3. The only change that was present was an increase in the KDR+CD133+ EPCs in the third visit when compared to baseline (p<0.05). There were no other significant changes in any other measurements. The changes in the foot ulcer area during the 12-week study period showed significant correlation with the following baseline measurements: FMD (r=-0.39, p=0.05), oxygen saturation in the periwound area (r=0.45, p<0.05) and vascular cell adhesion molecule-1 (VCAM, r=0.39, p=0.051). In addition, changes in the foot ulcer area were associated with changes in the CD34^+^ during the first four weeks of the study (r=0.53, p<0.05). No other additional correlations were observed between changes in the ulcer area and changes in the measured cytokines and growth factors. 

**Table 3:** Sequential flow cytometry, growth factor and cytokine measurements during the four study visits in the group of patients with DFU.

	**Visit 1 (Baseline)**	**Visit 2 (4 weeks)**	**Visit 3 (8 weeks)**	**Visit 4 (12 weeks)**
Ulcer size, % reduction from baseline	100	87 (52-131)	63 (20-112)	48 (18-127)
CD34^+^	172 (59 : 740)	314 (58 : 1129)	189 (45 : 963)	287 (61 : 1101)
CD34^+^KDR^+^	42 (20 : 175)	72 (20 : 185)	72 (28 : 207)	66 (18 : 243)
CD34^+^CD133^+^	61 (15 : 133)	39 (15 : 122)	43 (17 : 163)	70 (15 : 189)
KDR^+^CD133^+ a^	36 (10 : 93)	25 (11 : 153)	52 (21 : 104)	49 (17 : 161)
CD34^+^KDR^+^CD133^+^	3 (2 : 8)	6 (3 : 12)	5 (2 : 14)	8 (3 : 11)
Foot Endothelium Dependent Vasodilation (ACh response) (%)	20 (5 : 35)	12 (3 : 33)	22 (9 : 30)	19 (8 : 29)
Foot Endothelium Independent Vasodilation (SNP response) (%)	20 (6 : 51)	14 (7 : 36)	22 (8 : 34)	28 (12 : 41)
Oxy-Hb in peri-wound area (AU)	63 (56 : 74)	57 (47 : 73)	61 (46 : 82)	59 (52 : 80)
Deoxy-Hb in peri-wound area (AU)	47 (39 : 56)	45 (35 : 59)	47 (34 : 72)	48 (39 : 72)
O_2_ Hb Saturation in peri-wound area (%)	58 (54 : 59 )	57 (53 : 58)	56 (53 : 59)	55 (52 : 60)
Platelet-Derived Growth Factor-AA (ng/ml)	7.7 (4.5 : 13.7)	7.3 (5.0 : 11.8)	6.1 (4.0 : 11.6)	7.1 (4.3 : 13.3)
Platelet-Derived Growth Factor AB-BB (ng/ml)	50.9 (25.8 : 83.5)	45.7 (27.3 : 66.3)	38.4 (26.4 : 83.1)	45.0 (24.6 : 65.6)
EGF (pg/ml)	36.5 (14.0 : 99.3)	31.3 (18.7 : 74.0)	27.9 (17.0 : 43.8)	50.9 (8.2 : 71.2)
Fibroblast Growth Factor-2 (pg/ml)	20.0 (18.9 : 38.2)	16.2 (12.1 : 28.7)	22.9 (11.4 : 25.3)	18.9 (12.1 : 31.0)
VEGF (pg/ml)	185 (103 : 253)	185 (114 : 228)	211 (74 : 353)	154 (86 : 382)
Insulin (pg/ml)	468 (256 : 957)	710 (304 : 39805)	678 (404 : 2353)	601 (242 : 2088)
Leptin (ng/ml)	13.1 (7.3 : 30.5)	15.8 (10.3 : 24.3)	14.1 (8.6 : 17.8)	13.1 (9.1 : 24.7)
G-CSF (pg/ml)	32.7 (25.2 : 43.5)	33.9 (27.7 : 38.8	29.4 (23.6 : 40.4)	30.6 (19.4 : 43.7)
GM-CSF (pg/ml)	2.3 (1.5 : 3.5)	1.83 (0.91 : 3.36)	1.6 (1.1 : 2.5)	2 (1 : 3)
Eotaxin (pg/ml)	167 (107 : 202)	133 (93 : 207)	159 (73 : 206)	149 (108 : 212)
GRO (pg/ml)	840 (618 : 1261)	797 (542 : 1377)	1027 (580 : 1281)	853 (564 : 1203)
IFN γ (pg/ml)	2.45 (1.72 : 5.49)	3.06 (1.45 : 12.09)	2.70 (1.09 : 8.35)	1.94 (1.22 : 5.90)
IL-1α (pg/ml)	4.5 (1.9 : 11.2)	15.7 (6.0 : 34.0)	3.3 (1.9 : 6.0)	3.7 (2.5 : 6.2)
Interleukin-6 (pg/ml)	7.15 (3.61 : 13.90)	4.02 (2.15 : 8.55)	2.5 (1.2 : 14.0)	3.4 (1.3 : 6.4)
IL-8 (pg/ml)	29.5 (17.1 : 57.6)	17.7 (11.2 : 29.1)	29.4 (20.0 : 54.7)	22.2 (8.5 : 52.4)
Monocyte Chemoattractant Protein-1 (pg/ml)	605 (444 : 784)	722 (410 : 884)	531 (436 : 746)	638 (480 : 1012)
MDC (pg/ml)	1667 (1370 : 1962)	1784 (1515 : 2168)	1730 (1473 : 1963)	1897 (1474 : 2645)
TNFα (pg/ml)	14.6 (10.8: 22.0)	15.5 (8.2 : 18.9)	14.7 (8.5 : 18.2)	17.2 (11.2 : 20.6)
MMP-9 (ng/ml)	377 (272 : 525)	339 (213 : 555)	306 (199 : 403)	337 (252 : 622)
E-Selectin (ng/ml)	38.9 (26.2 : 55.1)	45.8 (27.2 : 60.7)	39.6 (26.4 : 49.9)	50.4 (31.9 : 68.6)
ICAM (ng/ml)	141 (109 : 170)	141 (105 : 177)	130 (101 : 156)	143 (118 : 174)
VCAM (ng/ml)	1202 (1016 : 1636)	1308 (1005 : 1811)	979 (851 : 1223)	1191 (967 : 1404)
CRP (μg/ml)	13.0 (5.0 : 53.7)	11.0 (4.7 : 82.5)	25.0 (12.2 : 52.2)	16.0 (4.3 : 45.9)

As the data was nonparametrically distributed the Kruskal - Wallis test was employed.

Median (25-75 interquartiles), flow cytometry results are presented as events/10^6^

a: visit 1 vs visit 3.

### Forearm skin biopsies

We evaluated the expression of SDF-1 and its receptor CXCR4 in the forearm skin biopsies taken from a different cohort of subjects that participated in another study. We evaluated biopsies from 12 healthy control subjects and 57 DM patients (10 non-neuropathic and 47 neuropathic). As there were no differences between non-neuropathic and neuropathic patients, all DM patients were evaluated as one group.

**Figure 4:**
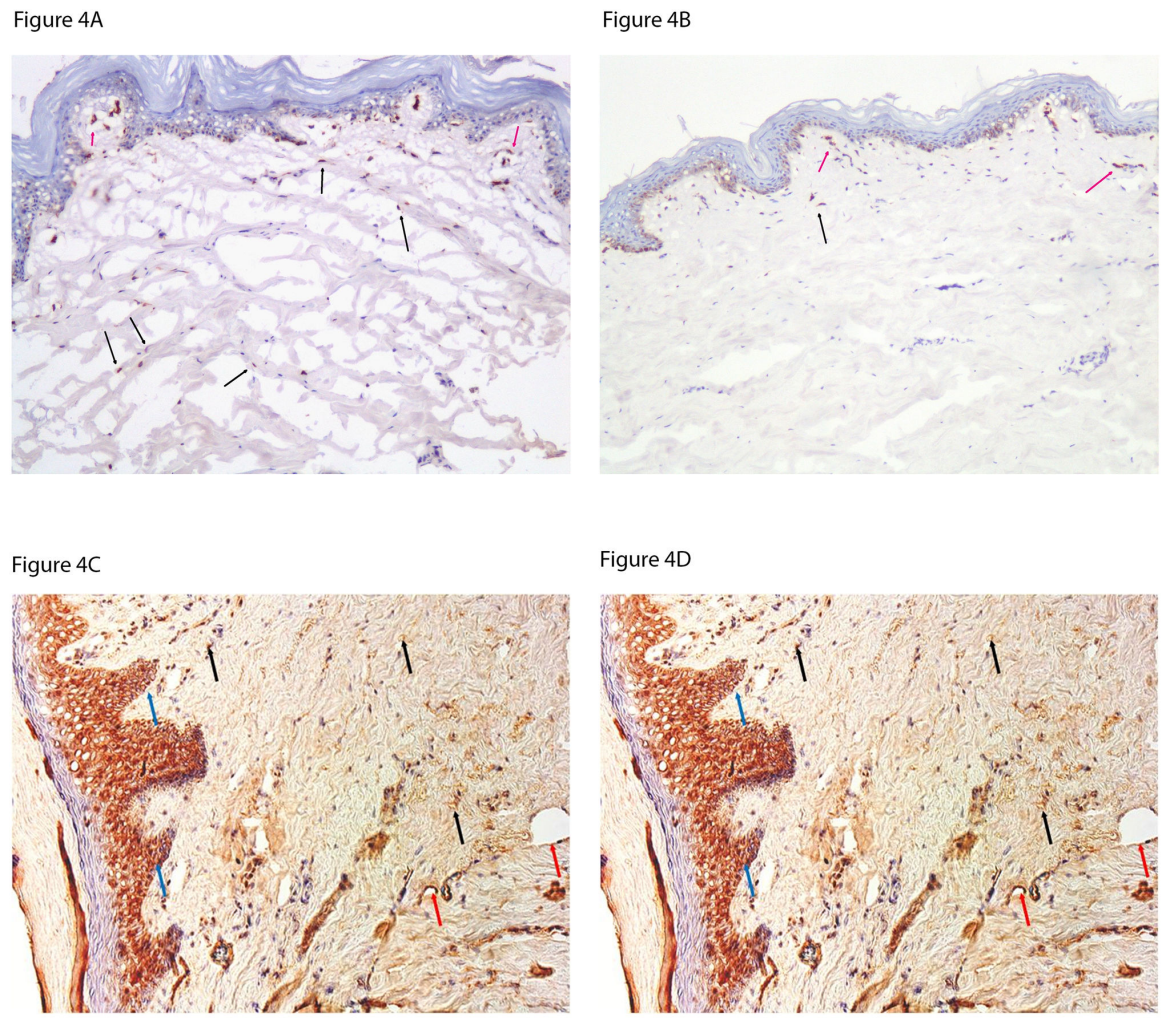
A and B: Forearm skin biopsy staining for SDF-1in a diabetic patient (Figure 4A) and a healthy control subject (Figure 4B), (frozen sections, x100). SDF-1 was expressed by stromal cells (black arrows) and endothelial cells (red arrows) and the staining pattern was mostly cytoplasmic and occasionally nuclear in cases of increased expression. The number of stained stromal cells and the intensity of staining were increased in in diabetic patients while no difference was found in the number of stained endothelial cells. *C* and *D*: Foot skin staining for CXCR4 in a diabetic patient (Figure 4C) and a healthy control subject (Figure 4D) (frozen sections, x200). CXCR4 was expressed by stromal cells (black arrows), endothelial cells (red arrows) and epithelial cells (blue arrows) and the staining pattern was mostly membranar and cytoplasmic. The intensity of staining was higher in in the diabetic group (p<0.05) but no differences were observed between the two groups in the number of positive stromal and endothelial cells (p=NS).

SDF-1 was expressed by stromal and endothelial cells (Figures 4A and 4B). The number of SDF-1 postive stromal cells in the dermis was higher in DM patients when compared to the control subjects (2.2±0.7 vs 1.7±0.6, p<0.05) while no differences were observed in endothelial cells (2.3±0.6 vs 2.0 ± 1.0, p=NS). In addition to the number of cells, the intensity of staining was also higher in the DM patients (3.1±0.7 vs 2.5±0.8, p<0.01).

There were no differences in the number of CXCR4 positive stromal cells (1.9±0.6 vs 1.7 ±0.6, p=NS) or the intensity of staining (2.5 ±0.8 vs 2.5±0.8, p=NS) between the two groups. However its expression tended to be lower in the endothelial cells of diabetic patients (2.1 ±0.6 vs 2.2±0.6, p=0.069). 

### Foot skin biopsies

The SDF-1 staining pattern was similar to the one observed in the forearm biopsies. No differences were observed between the two groups in the number of positive stromal cells, endothelial cells or the intensity of staining (p=NS). The intensity of CXCR4 staining (Figures 4C and 4D) was higher in the DM group (3±1 vs 4±0, p<0.05) but there were no differences in the number of positive stromal and endothelial cells (p=NS) between the two groups.

### Animal Models

Both diabetic mice and rabbits had impaired wound healing ten days post-wounding when compared to their respective controls (p<0.05). The results of the flow cytometry studies in both models are shown in Table 4. In general, in both models at both study timepoints (baseline, before wounding, and ten days post-wounding), all tested phenotypes of EPCs tended to be higher in diabetic animals when compared to their non-diabetic counterparts. In the mouse model, the tested phenotypes showed a tendency to increase ten days post-wounding in both diabetic and non-diabetic animals. In contrast, rabbits tended to have lower EPC counts after wound creation in both the diabetic and non-diabetic state but, in most cases, this reduction failed to reach statistical significance.

**Table 4:** Flow cytometry results in the non-diabetic and diabetic mice and rabbit animal models before and ten days after wounding.

	**C57BL/6 Mice Before Wounding(CMB, n=34)**	**C57BL/6 Mice After Wounding(CMW, n=7**)	**STZ-Diabetic C57BL/6 Mice Before Wounding(DMB, n=11)**	**STZ-Diabetic C57BL/6 Mice After Wounding(DMW, n=21)**
CD34^+^FLK-1^+ a^	20 (0:81)	40 (10:220)	25 (10:150)	270 (130:600)
CD34^+^CD133^+ b^	0 (0:10)	30 (10:40)	10 (0:89)	40 (27:69)
FLK-1^+^CD133^+^	80 (20:206)	140 (50:270)	128 (10:310)	210 (100:384)
CD34^+^FLK-1^+^CD133^+ c^	0 (0:13)	30 (10:40)	9 (0:50)	70 (64:150)
	**Rabbits Before Wounding(CRB, n=8)**	**Rabbits After Wounding(CRW, n=7**)	**Alloxan-Diabetic Rabbits Before Wounding(DRB, n=8)**	**Alloxan-Diabetic Rabbits After Wounding(DRW, n=13)**
CD34^+^	132 (102:225)	146 (30:558)	665 (86:1956)	427 (249:3469)
CD34^+^KDR^+ d^	105 (55-163)	87 (39:272)	669 (110:1246)	273 (124:1134)
CD34^+^CD133^+ e^	0 (0:4)	4 (0:25)	63 (12:249)	39 (4:659)
KDR^+^CD133^+ f^	172 (108:251)	127 (58:152)	439 (359:887)	580 (59:1105)
CD34^+^KDR^+^CD133^+ g^	28 (2:35)	6 (0:24)	103 (12:238)	51 (10:167)

As the data was nonparametrically distributed the Kruskal - Wallis test was employed.

Flow cytometry results are presented as events/10^6^

a: CMB vs DMB: p <0.05, DMB vs DMW: p<0.01; b: CMB vs CMW: p<0.05; c: CMB vs CMW: p <0.05, DMB vs DMW: p<0.01, CMW vs DMW: p<0.01; d: CRW vs DRW: p<0.05; e: CRB vs DRB: p<0.05, CRW vs DRW: p<0.05; f: CRB vs DRB: p<0.05, CRB vs CRW: p<0.05; g: CRB vs DRB: p<0.05, CRW vs DRW: p<0.05.

In the mouse model, when all animals were considered as one group, positive correlations were observed at baseline between FLK-1^+^CD133^+^ and serum levels of ICAM (r=0.44, p<0.05), VCAM (r=0.68, p<0.001) and tissue plasminogen activator-1 (tPA-1, r=0.52, p<0.05), CD34^+^CD133^+^ and ICAM (r=0.43, p<0.05), VCAM (r=0.71, p<0.001) and tPA-1 (r=0.44, p<0.05) and CD34^+^FLK-1^+^ and ICAM (r=0.44, p<0.05), VCAM (r=0.70, p<0.001) and tPA-1 (r=0.45, p<0.05). 

In non-diabetic rabbits, at baseline, strong correlations were observed between KDR^+^CD133^+^ and serum levels of interferon gamma (IFNγ, r= -0.58, p<0.01), IL-8 (r=0.56, p<0.01) and macrophage-derived chemokine (MDC, r=-0.43, p<0.005), CD34^+^CD133^+^ and GM-CSF (r=0.48, p<0.05) and IL-8 (r=0.56, p<0.01) and CD34^+^KDR^+^ and IL-8 (r=0.47, p<0.05). No asociations were observed in the diabetic animals.

## Discussion

The main findings of the current study are: EPCs were reduced in diabetic patients with complications and the presence of DFU had no additional effect; compared to all the other groups, DFU was associated with higher levels of numerous inflammatory cytokines at baseline while complete healing was associated with lower CRP, IL-1α and GM-CSF levels at baseline; serum SDF-1 levels were increased in DFU patients, its expression in forearm skin biopsies was increased in diabetic patients while no major changes were observed in the expression of its receptor CXCR4 at both forearm and foot skin specimens; complete wound healing was associated with a parallel reduction in circulating CD34^+^KDR^+^ cells suggesting enhanced homing of these cells during the healing process; diabetic mice and rabbits that have impaired wound healing also had increased EPCs ten days after wounding when compared to non-diabetic animals, suggesting impaired trafficking of the progenitor cells.

Previous studies in our unit have shown that conditions that precede the development of DFU, such as increased inflammation and aberrant secretion of serum growth factors, are associated with impaired wound healing [3]. In the present study, we focused on the factors that are associated with the healing of an existing DFU. We first focused on EPCs as they are known to play an important role in wound healing [16] and are affected by diabetes, especially in the presence of macrovascular disease [17]. 

There is currently no consensus about the characteristics of EPCs and the common thought is that they are comprised of heterogeneous subpopulations that express various antigens [18]. The most currently used phenotypic markers are hematopoietic (CD34), early hematopoietic stem-cell (CD133) and endothelial [VEGR receptor 2 (also known as KDR in human and FLK-1 in mice)] [19,20]. In the current study we have measured all of these phenotypic markers, including CD34^+^ that according to some reports are also characterized as circulating endothelial cells (CECs) [21]. Although none of the study subjects had macrovascular disease, our results indicate that patients at risk of DFU and/or with DFU had reduced counts of most of the studied EPC phenotypes (Table 2). The only difference in EPC measurements between patients at risk of DFU and patients with DFU was that there were reduced numbers of CD34^+^KDR^+^ cells, in the patients at risk of DFU. These results indicate that the number of circulating EPCs is not related to the presence of diabetes but the presence of neuropathy. Furthermore, in agreement with previous studies [22], EPCs were associated with FMD, indicating their role in maintaining endothelial function. However, the rather low correlation coefficient r between FMD and all EPC phenotypes indicates a rather weak association and clearly suggests that there are other factors that affect both measurements. Finally, in contrast with previous studies [23], within the DM patients, no correlations were observed between EPC and HbA1c, clearly indicating that the main factor that influenced EPC numbers was the presence of diabetic complications.

Furthermore, we found that the baseline numbers of all EPC phenotypes were not predictive of complete healing or reduction of the ulcer size during the 12-week study period (Table 3). However, patients whose ulcers healed had reduced CD34^+^KDR^+^ at visits 3 and 4 (Figure 2). *In vivo* the CD34^+^KDR^+^ phenotype is known to promote endothelial cell proliferation and neovascularization and is considerd as one of the most represented EPC phenotypes [24]. CD34^+^KDR^+^ cells were increased in DFU patients when compared to at risk of DFU patients (Table 2) and were reduced at the end of the study in patients who healed their ulcers (Figure 2). Thus, it can be hypothesized that wound healing requires early release of CD34^+^KDR^+^ cells from the bone marrow while the subsequent observed reduction could be associated with homing of these cells to the ulcer area. However, although this hypothesis is in agreement with animal studies [25], it cannot be proven by the current study design and will need further investigation. Of interest, an increase in the same phenotype has been associated with a reduced risk of death from cardiovascular causes [5].

SDF-1 plays an important role in angiogenesis and wound healing. In conjunction with vascular endothelial growth factor (VEGF)-A, it mobilizes EPCs from the bone marrow while through its interaction with its receptor CXCR4 promotes the homing of the EPCs to the wound area [26]. Although animal studies have shown reduced SDF-1 expression in diabetic cutaneous wounds and SDF-1 treatment has shown to reverse the diabetic defect in EPC homing in diabetic mice [26], there is limited information in DM patients. More recently, inhbition of SDF-1 signaling has been linked to impaired healing by decreasing cellular migration and angiogenesis, modulation of inflammatory cytokines and inflammation [27].Our data indicates that serum SDF-1 levels were not affected by diabetes or neuropathy but were increased in DM patients with DFU (Table 2). A similar increase was also observed in the serum stem cell factor (SCF) that is released by matrix metalloproteinase-9 (MMP-9) and permits the transfer of endothelial and hematopoietic stem cells from the quiescent to proliferative niche (Table 2) [28]. Furthermore, in contrast to animal studies [26], no reductions were observed in the forearm and foot skin SDF-1 expression of DM patients while the SDF-1 expression was higher in forearm stromal cells (Figure 4). However, baseline SDF-1 and SCF serum levels were not associated with complete wound healing or ulcer size reduction during the study period. A recent study that reported increased levels of colony-forming units (CFU)-Hill EPCs in type 1 DM patients with non-proliferative retinopathy found no changes in serum SDF-1 levels [20]. Finally, no changes were observed in the forearm and foot skin CXCR4 expression that is also involved in wound healing [29]. 

DFU patients had higher levels of a number of inflammatory cytokines, including IL-8, TNFα and CRP (Table 2) but only lower levels of CRP were associated with complete wound healing during the study (Figure 3A). CRP is an acute-phase protein and its synthesis by the liver is rapidly dysregulated in various conditions, including tissue damage and infection [30]. Our data suggest that local factors that affect wound healing, such as tissue necrosis and infection may be better reflected by the systemic CRP levels than any other inflammatory cytokine. In addition, patients who healed their ulcers had lower IL-1α at both the baseline and exit study visits (Figure 3B). IL-1α, an acute-phase epidermal cytokine constitutively produced by keratinocytes, and also produced by fibroblasts, macrophages, and granulocytes, acts synergistically with TNF-α in promoting inflammation [31]. GM-CSF, a proinflammatory cytokine that is involved in inflammation, infection, production of granulocytes and macrophages and on that has been used for the treatment of chronic wounds [32] was increased at baseline (Figure 3C). Our results are compatible with changes that were observed in inflammatory cytokines in biopsies of chronic venous ulcers that were collected before and after four weeks of compression therapy [33]. Confirmation of these results may lead to the use of CRP, IL-1α and GM-CSF as prognostic markers for DFU healing.

The reduction in the DFU area during the study was associated with FMD and serum sVCAM levels but not with any measurement of the foot skin microvascular reactivity. These results indicate that systemic changes are more important than local changes in endothelial function for wound healing. Finally, in contrast with previous studies, measurements of foot oxy and deoxy-hemoglobin did not differ any of the groups and were not associated with ulcer healing [14,34]. 

Various animal models of diabetic wound healing have been tested as none of them is satisfactorily representative of the human condition [15]. We studied EPC changes using the same EPC markers as in the human study in diabetic mice and rabbits before and after the wound creation. Our results showed no EPC reduction in both diabetic animal models in either study timepoints, before or ten days after wounding (Table 4). Given that both animal models were devoid of any serious complications, these results are consistent with the lack of differences between healthy controls and diabetic patients without complications and further emphasize the role of diabetic complications in EPC measurements. Our results are consistent with a previous study that reported no EPC changes before and after wounding in mice with diabetes of four week duration [35]. However, other studies that employed different markers [26,36] have reported reduced circulating EPC in diabetic mice, further emphasizing the need to compare similar markers in both human and animal studies. In addition, EPCs in both non-diabetic and diabetic rabbits tented to be lower ten days post- wounding when compared to baseline, while these cells tended to be higher in diabetic mice, indicating differences between the two models with the rabbits being more representative to human changes [15]. In agreement with the human studies, mouse EPCs showed positive correlations with markers of endothelial function, such as ICAM and VCAM, raising the question whether impaired endothelial function prevented homing. On the other hand, EPCs of non-diabetic rabbits positively correlated with factors such as GM-CSF and IL-8 that are known to influence EPC mobilization in humans [37]. These results further emphasize the fact that no model is completely satisfactory in representing the human condition and the need to use multiple animal models.

The present study has its limitations. The healing rate in the DFU group was rather small, indicating that we included patients with chronic wounds and limited healing capacity. However, we believe that, as meaningful comparisons between healers and non-healers were observed, the size of the group did not affect the extraction of reliable conclusions. In addition, although both groups with diabetic neuropathy tended to have higher creatinine levels, we excluded patients with end stage renal failure. This, in conjunction with the exclusion of patients with macrovascular disease, drastically reduces the possibility that renal or macrovascular complications were important confounding factors in the observed results. Finally, we did not apply statistical correction for multiple testing for the cytokine measurements. The main reason is that cytokines are influenced by the same factors and cannot be considered as independent of each other. As a result, we believe that applying statistical corrections in this study is not appropriate and would carry a high potential of reporting erroneous false negative results since this is not a clinical trial but rather a study that focuses on the pathophysiology of wound healing.

In summary, our results indicate that EPCs are reduced in diabetic patients at risk of DFU or patients with active DFU. In addition, complete wound healing is associated with parallel EPC reduction, suggesting possible increased homing. Baseline CRP, IL-1α and GM-CSF were associated with complete wound healing and may be helpful as prognostic markers of DFU healing. Diabetic mice and rabbits do not sufficiently represent the human EPC changes that occur during diabetic wound healing, indicating the need of using multiple animal models. 
